# A sensitive and specific histopathologic prognostic marker for *H3F3A* K27M mutant pediatric glioblastomas

**DOI:** 10.1007/s00401-014-1338-3

**Published:** 2014-09-09

**Authors:** Sriram Venneti, Mariarita Santi, Michelle Madden Felicella, Dmitry Yarilin, Joanna J. Phillips, Lisa M. Sullivan, Daniel Martinez, Arie Perry, Peter W. Lewis, Craig B. Thompson, Alexander R. Judkins

**Affiliations:** 1 Cancer Biology and Genetics Program, Memorial Sloan Kettering Cancer Center (MSKCC), New York, NY 10065 USA; 2 Department of Pathology and Laboratory Medicine, Children’s Hospital of Philadelphia, Perelman School of Medicine, University of Pennsylvania, Philadelphia, PA USA; 3 Department of Pathology, Henry Ford Health System, Detroit, MI USA; 4 Molecular Cytology Core Facility, MSKCC, New York, NY USA; 5 Department of Pathology, University of California, San Francisco, CA USA; 6 Department of Biomolecular Chemistry, University of Wisconsin-Madison, Madison, USA; 7 Department of Pathology and Laboratory Medicine, Children’s Hospital Los Angeles, Keck School of Medicine University of Southern California, 4650 Sunset Boulevard #43, Los Angeles, CA 90027 USA

**Keywords:** Pediatric glioblastoma, *H3F3A* mutation, K27M mutation, H3K27me3, Diagnostic biomarker, Prognosis, Methylation, Epigenetics

## Abstract

Pediatric glioblastomas (GBM) are highly aggressive and lethal tumors. Recent sequencing studies have shown that ~30 % of pediatric GBM and ~80 % of diffuse intrinsic pontine gliomas show K27M mutations in the *H3F3A* gene, a variant encoding histone H3.3. *H3F3A* K27M mutations lead to global reduction in H3K27me3. Our goal was to develop biomarkers for the histopathologic detection of these tumors. Therefore, we evaluated the utility of measuring H3K27me3 global reduction as a histopathologic and prognostic biomarker and tested an antibody directed specifically against the H3.3 K27M mutation in 290 samples. The study cohort included 203 pediatric (including 38 pediatric high-grade astrocytomas) and 38 adult brain tumors of various subtypes and grades and 49 non-neoplastic reactive brain tissues. Detection of H3.3 K27M by immunohistochemistry showed 100 % sensitivity and specificity and was superior to global reduction in H3K27me3 as a biomarker in diagnosing *H3F3A* K27M mutations. Moreover, cases that stained positive for H3.3 K27M showed a significantly poor prognosis compared to corresponding negative tumors. These results suggest that immunohistochemical detection of H3.3 K27M is a sensitive and specific surrogate for the *H3F3A* K27M mutation and defines a prognostically poor subset of pediatric GBM.

**Electronic supplementary material:**

The online version of this article (doi:10.1007/s00401-014-1338-3) contains supplementary material, which is available to authorized users.

## Introduction

Genomic sequencing has substantially enhanced the understanding of the genetics and molecular biology of brain tumors. Many new driver mutations have been identified that will help in elucidating the pathogenesis of these disorders. Consequently, this has led to a shift in the paradigm of how we approach the diagnosis of brain tumors, where genomic information is integral and complementary to histopathologic diagnoses.

Sequencing studies of pediatric high-grade gliomas [including glioblastomas, anaplastic astrocytomas and diffuse intrinsic pontine gliomas (DIPG)] have shown recurrent mutations in the *H3F3A* gene (encoding the histone H3.3). Mutations were noted at position 27, where the lysine residue is replaced by methionine (K27M) or at position 34, the glycine residue is replaced by arginine or valine (G34R/V) [3, 4, 9, 10, 13, 17, 18, 24]. A small percentage of DIPG cases also showed *HIST1H3B* encoding the histone H3.1 where the lysine residue at position 27 is replaced by methionine (K27M) [3, 24]. Since lysine residues on histone tails are subject to post-translational modifications, we and others have shown that the *H3F3A* K27M mutation results in global reduction in H3K27me3 [2, 6, 15, 22]. H3K27 is trimethylated by the polycomb repressive complex 2 (PRC2) to generate the repressive H3K27me3 mark. Enhancer of zest 2 (EZH2) is the main methyltransferase enzyme contained in this complex that is responsible for trimethylation of H3K27. The H3 K27M mutant protein binds to EZH2 to inhibit its methyltransferase activity, resulting in a global reduction of H3K27me3 [2, 15]. Moreover, these tumors also show a DNA hypomethylation phenotype [2, 12, 18]. The mechanisms by which *H3F3A* mutations contribute to the pathogenesis of gliomas are not clearly understood and are currently being investigated.

Current methodology of assessing these mutations is based on sequencing studies. While time consuming and tedious, this methodology may not be readily accessible to all laboratories. Thus, we sought to evaluate histopathologic biomarkers for evaluating *H3F3A* K27M mutations to aid in establishing the diagnosis of these tumors using immunohistochemistry (IHC), a tool that is readily available to most pathology practices. To this end, we assessed if detection of global reduction of H3K27me3 by immunohistochemical staining could be used as a molecular surrogate for *H3F3A* K27M mutations. We compared global reduction in H3K27me3 with a novel antibody specifically targeting H3.3 K27M mutations. Western blot analysis showed that the antibody raised against the N-terminal of the H3.3 K27M protein was specific for the mutation [15]. We used IHC to detect overall levels of H3K27me3 in 290 samples consisting of pediatric brain tumors, adult brain tumors and non-neoplastic brain tissues. Our goal was to determine the sensitivity and specificity of both markers in diagnosing *H3F3A* K27M mutations. Our results indicate that antibody directed against H3.3 K27M is superior to global reduction of H3K27me3 as a marker in diagnosing *H3F3A* K27M mutant GBM with 100 % sensitivity, specificity and positive and negative predictive values and can be used as a prognostic indicator.

## Methods

### Cohort

Tissue samples were obtained from the Departments of Pathology and Laboratory Medicine from Children’s Hospital of Philadelphia, Children’s Hospital Los Angeles and the University of San Francisco after institutional review board approval from all institutions. All identifiers from cases were removed prior to analysis. 241 cases were contained in previously well-characterized tissue microarrays (TMA) and only tissue cores containing more than 95 % tumor were included in this analysis [5, 19, 21–23]. Pediatric brain tumors consisted of 36 pilocytic astrocytomas (Pilo, WHO grade I), 5 subependymal giant cell astrocytoma (SEGA, grade I), 9 dysembryoplastic neuroepithelial tumors (DNET, grade I), 17 gangliogliomas (GG, grade I), 16 grade II astrocytomas (Astro, 14 fibrillary astrocytomas, 1 pilomyxoid astrocytoma, and 1 pleomorphic xanthoastrocytoma, all grade II), 5 oligodendrogliomas (Oligo, grade II), 10 meningiomas (Men, grade I), 24 medulloblastomas (MB, grade IV) 26 atypical teratoid/rhabdoid tumors (AT/RT, grade IV), 3 craniopharyngiomas (CP, grade I), 11 choroid plexus papillomas (CPP, grade I), 3 neurocytomas (NC, grade II) and 38 high-grade astrocytomas including 4 anaplastic astrocytomas (AA, grade III) and 34 glioblastomas (GBM, grade IV) (collectively referred to as pediatric GBM for simplicity). Of the 203 pediatric tumors, we have previously reported H3K27me3 in 40 cases including 20 pediatric GBMs [22]. Adult tumors consisted of 4 pilocytic astrocytomas (PA, grade I), 2 gangliogliomas (GG, grade I), 3 diffuse astrocytomas (DA, grade II), 8 anaplastic astrocytomas (AA, grade III), 3 oligodendrogliomas (OD, grade II), 5 anaplastic oligodendrogliomas (AO, grade III) and 13 glioblastomas (GBM, grade IV). The demographics of these cases have been reported elsewhere [5, 19, 21–23]. Non-neoplastic tissues were studied as full slides and included normal brain (*n* = 3), vascular malformation (*n* = 4), ischemia (*n* = 4), hemorrhage (*n* = 5), CNS malformations (*n* = 5), infectious disease (*n* = 8), vasculitis (*n* = 5), hippocampal sclerosis (*n* = 5), demyelinating disorders (*n* = 5) and metastatic tumors (*n* = 5). Two independent neuropathologists reviewed all cases in a blinded manner.

### Immunohistochemistry and automated scoring

Immunohistochemical studies were performed as previously published [22]. In brief, immunostaining was performed using the Discovery XT processor (Ventana Medical Systems). A commercially available rabbit polyclonal antibody that recognizes the N-terminus of Histone H3.3, K27M mutant protein (anti-H3 K27M, #ABE419, Millipore, Billercia, MA, USA; 0.5 μg/mL) was used [15]. To detect H3K27me3, a rabbit polyclonal anti-H3K27me3 (07-449, Millipore, Billercia, MA, USA; 1 μg/mL) was used as previously described [22]. Tissue sections were blocked for 30 min in 10 % normal goat serum in 2 % BSA in PBS. Sections were incubated for 5 h with the anti-H3K27me3 or anti-H3.3 K27M antibodies. Tissue sections were then incubated for 60 min with biotinylated goat anti-rabbit IgG (Vector labs, PK6101) at 1:200 dilution. Blocker D, Streptavidin-HRP and DAB detection kit (Ventana Medical Systems) were used according to the manufacturer instructions. To ensure uniformity of IHC, we compared staining in 40 cases contained in tissue microarrays with their corresponding full sections (40 randomly selected cases including 20 pediatric GBM) selected from the same blocks that were used to generate the TMA for both H3K27me3 and H3.3 K27M. For both markers, staining results were identical in tissue microarrays and full sections. Additionally, we evaluated both H3K27me3 and H3.3 K27M in full sections for all 38 cases of pediatric high-grade astrocytomas.

H3K27me3 and H3.3 K27M stained tumor sections were scored/quantified by automated image processing software (data are presented in Fig. 2). Automated scoring was performed by scanning each slide using a Pannoramic Flash 250 scanner (Perkin Elmer, Waltham, MA, USA). Scanned slides were viewed through the Pannoramic viewer software program (3D Histech, Waltham, MA, USA). An individual blinded to the experimental design captured JPEG images from each core or full section (circular area of 315 cm^2^ corresponding to the entire core or randomly chosen equivalent area from full sections) at 10× magnification on the Pannoramic viewer software program. Quantification of immunostaining on each JPEG was conducted using an automated analysis program with Matlab’s image processing toolbox based on previously described methodology [22]. The algorithm used color segmentation with RGB color differentiation, *K*-means clustering and background–foreground separation with Otsu’s thresholding. To arrive at a score the number of extracted pixels was multiplied by their average intensity for each core. The final score for a given case and marker was calculated by averaging the score of two randomly chosen areas for each full section or two core tumor samples or for each case.

### *H3F3A* gene sequencing

Sanger sequencing for *H3F3A* coding exons was performed as previously described [22]. Paraffin-embedded formalin-fixed (FFPE) blocks from GBMs were used to extract genomic DNA was extracted from two 10 μ slices. The Formapure kit (Agencourt, Beverly, MA, USA) was used according to manufacturers’ method in a 96-well format in a semi-automated fashion using primers previously standardized [22]. M13 tails were added to facilitate Sanger sequencing. PCR reactions were set up in 384 well plates, in a Beckman Coulter Biomek^®^ FX, and run in a Duncan DT-24 water bath thermal cycler, with 10–40 ng of genomic DNA as template, HotStart Taq (Kapa Biosystems, Woburn, MA, USA), 250 μM dNTPs, 1× PCR buffer, 6 % DMSO and 0.2 μM primers. A “touchdown” PCR method was used, which consisted of: 1 cycle of 95 °C for 5 min; 3 cycles of 95 °C for 30 s, 64 °C for 30 s, 72 °C for 60 s; 3 cycles of 95 °C for 30 s, 62 °C for 30 s, 72 °C for 60 s; 3 cycles of 95 °C for 30 s, 60 °C for 30 s, 72 °C for 60 s; 37 cycles of 95 °C for 30 s, 58 °C for 30 s, 72 °C for 60 s; 1 cycle of 70 °C for 5 min. Amplified DNA was purified using AMPure (Agencourt Biosciences, Beverly, MA, USA). The purified PCR reactions were split into two, and sequenced bidirectionally with M13 forward and reverse primer and Big Dye Terminator Kit v.3.1 (Applied Biosystems, Foster City, CA, USA), at Agencourt Biosciences. Dye terminators were removed using the CleanSEQ kit (Agencourt Biosciences), and sequence reactions were run on ABI PRISM 3730xl sequencing apparatus (Applied Biosystems, Foster City, CA, USA). The sequences were analyzed using an automated mutation detection pipeline developed by the MSKCC Bioinformatics Core.

### Statistical analysis

Statistical analyses were performed in consultation with the Memorial Sloan-Kettering biostatistics core. SPSS (version 21, IBM, Chicago, IL, USA) and Prism (version 5, La Jolla, CA, USA) were used to analyze data. All statistical tests were two sided. Statistical significance was set at the 0.05 level. Student’s *t* test was used to compare H3K27me3 or H3.3 K27M levels between *H3F3A* K27M mutant and wild-type tumors. The log-rank (Mantel–Cox) test was used to examine the association of H3.3 K27M positivity with overall survival. Multivariate survival analysis was performed using the Cox proportional hazards model. Differences were considered significant when *p* < 0.05 (95 % confidence intervals).

Diagnostic performance measures including sensitivity, specificity, positive predictive value (PPV) and negative predictive value (NPV) for each marker were calculated using standard statistical methodology [16]. Sensitivity is defined as the probability of a positive test result (immunopositive) given that the subject has *H3F3A* *K27M* mutations. Specificity is defined as the probability of a negative test result (immunonegative) given that the sample does not bear the mutation. PPV is defined as the percentage of patients with a positive test (immunopositive) who actually bear *H3F3A* K27M mutations and NPV is defined as negative test (immunonegative) in patients who do not have the mutation.

## Results

### Assessment of H3K27me3 immunoreactivity in pediatric and adult brain tumors and non-neoplastic brain tissue samples

H3K27me3 was evaluated using IHC with automated scoring in a cohort of 241 brain tumors consisting of 203 pediatric and 38 adult brain tumors. The cohort consisted of 38 pediatric high-grade astrocytomas and we used Sanger sequencing as previously described to determine the histone mutational status in all these tumors [22]. Sanger sequencing revealed 12 *H3F3A* K27M mutant tumors and 1 *H3F3A* G34R tumor; no *HIST1H3B* mutations were detected (Supplementary Table S1). Of these 38 tumors, one *H3F3A* wild-type tumor recurred.

**Fig. 1:**
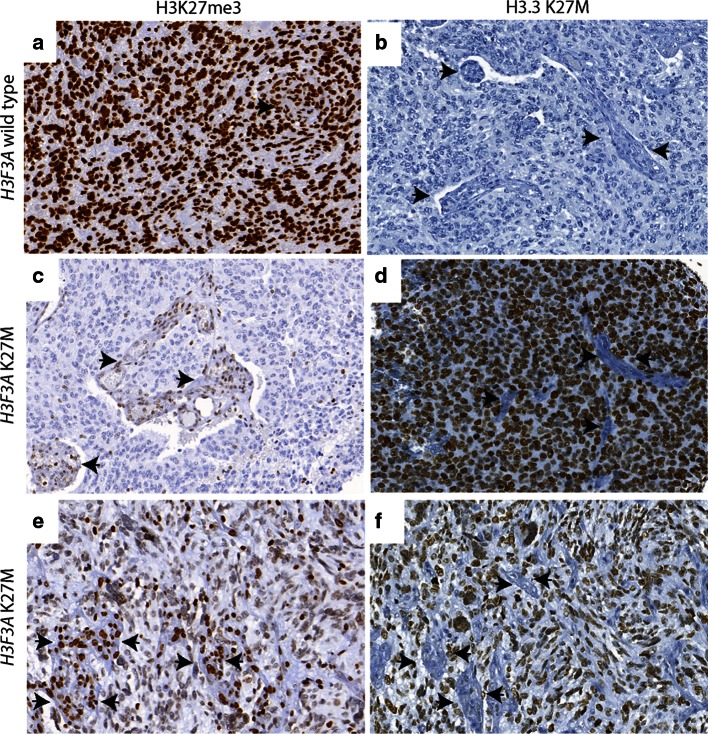
Comparison of immunostaining for H3K27me3 and H3.3 K27M in wild-type and *H3F3A* K27M mutant pediatric GBM. **a**, **b** Representative images from a *H3F3A* K27M wild-type tumor, H3K27me3 (40×, **a**) and H3.3 K27M (40×, **b**). **c**–**f**. Representative images from two different *H3F3A* K27M mutant tumors, H3K27me3 (40×, **c** and 60×, **e**) and H3.3 K27M (40×, **d** and 60×, **f**). *Arrowheads* indicate endothelial cells in blood vessels

**Fig. 2:**
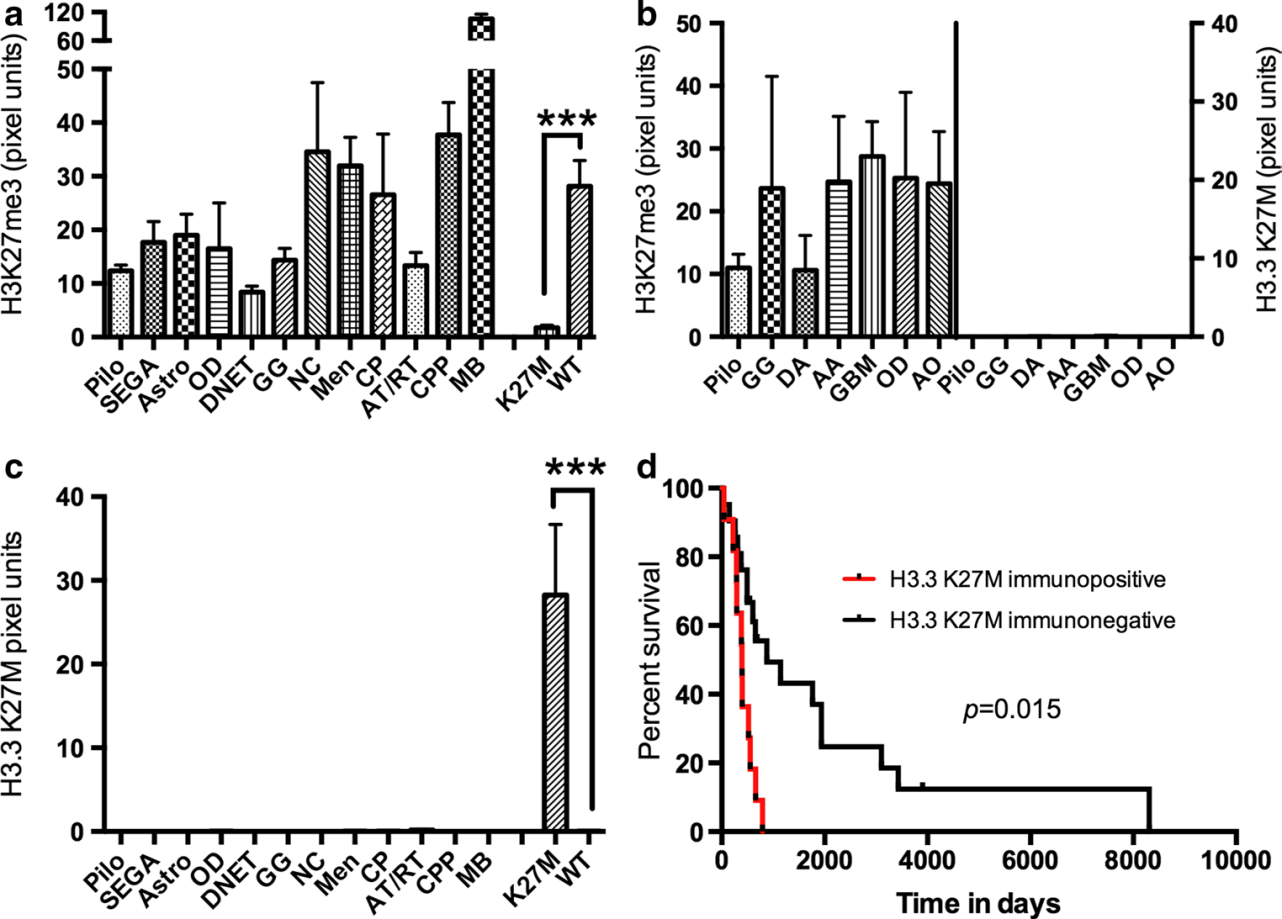
Quantification of H3K27me3 and H3.3 K27M immunostaining in adult and pediatric brain tumors and survival analyses in pediatric high-grade gliomas. **a**, **c** Quantification of H3K27me3 (**a**) and H3.3 K27M (**c**) IHC in pediatric brain tumors. ***p < 0.0001. *Pilo* pilocytic astrocytoma, *SEGA* subependymal giant cell astrocytoma, *Astro* grade II astrocytoma, *OD* oligodendroglioma, *DNET* dysembryoplastic neuroepithelial tumor, *GG* ganglioglioma, *NC* neurocytoma, *MEN* meningioma, *CP* craniopharyngioma, *AT/RT* atypical teratoid/rhabdoid tumor, *CPP* choroid plexus papilloma, *MB* medulloblastoma, *K27M* *H3F3A* K27M mutant GBMs, *WT* GBMs without *H3F3A* K27M mutations. **b** Quantification of H3K27me3 (*left Y-axis*) and H3.3 K27M (*right Y-axis*) in adult brain tumors. *Pilo* pilocytic astrocytoma, *DA* diffuse astrocytoma, *GG* ganglioglioma, *AA* anaplastic astrocytomas, *GBM* glioblastoma, *OD* oligodendroglioma, *AO* anaplastic oligodendrogliomas. **d** Kaplan–Meier survival analyses between H3.3 K27M immunopositive (*red line*) and H3.3 K27M immunonegative (*black line*) pediatric GBMs (see also Table 2)

**Fig. 3:**
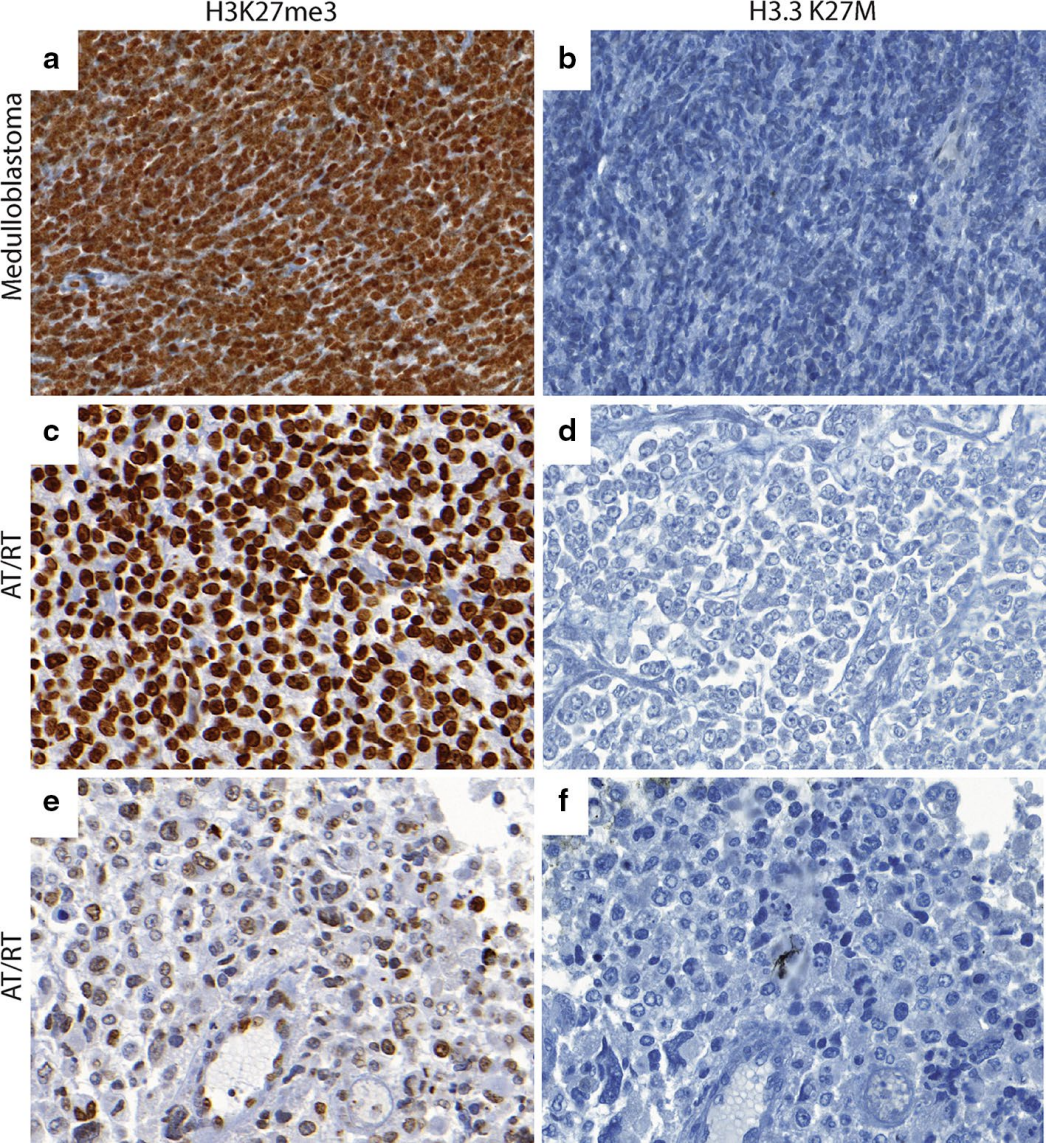
Comparison of H3K27me3 and H3.3 K27M in CNS embryonal tumors. **a**, **b** Representative images of H3K27me3 (40×, **a**) and H3.3 K27M (40×, **b**) in medulloblastoma. **c**, **d** Representative images of H3K27me3 (40×, **c**) and H3.3 K27M (40×, **d**) in an AT/RT case with high H3K27me3. **e** , **f** Representative images of H3K27me3 (40×, **e**) and H3.3 K27M (40×, **f**) in subset of AT/RT with low H3K27me3

**Fig. 4:**
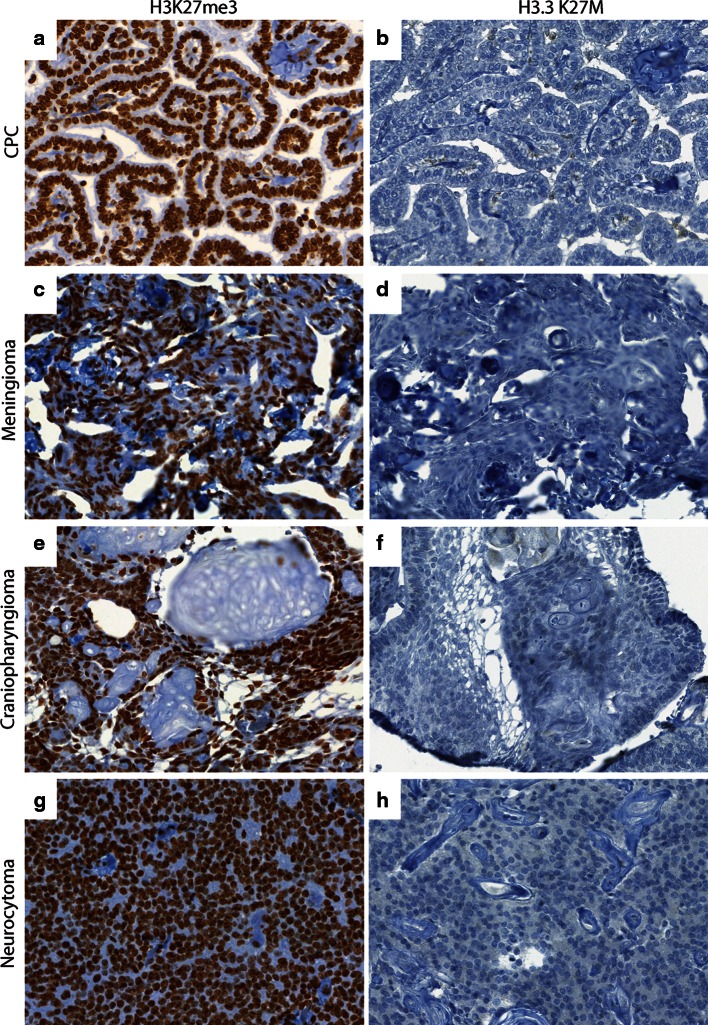
Comparison of H3K27me3 and H3.3 K27M in pediatric brain tumors. **a**, **b** Representative images of H3K27me3 (40×, **a**) and H3.3 K27M (40×, **b**) in choroid plexus papilloma (CPC). **c**, **d** Representative images of H3K27me3 (40×, **c**) and H3.3 K27M (40×, **d**) in meningioma. **e**, **f** Representative images of H3K27me3 (40×, **e**) and H3.3 K27M (40×, **f**) in craniopharyngioma. **g**, **h** Representative images of H3K27me3 (40×, **g**) and H3.3 K27M (40×, **h**) in neurocytoma

**Fig. 5:**
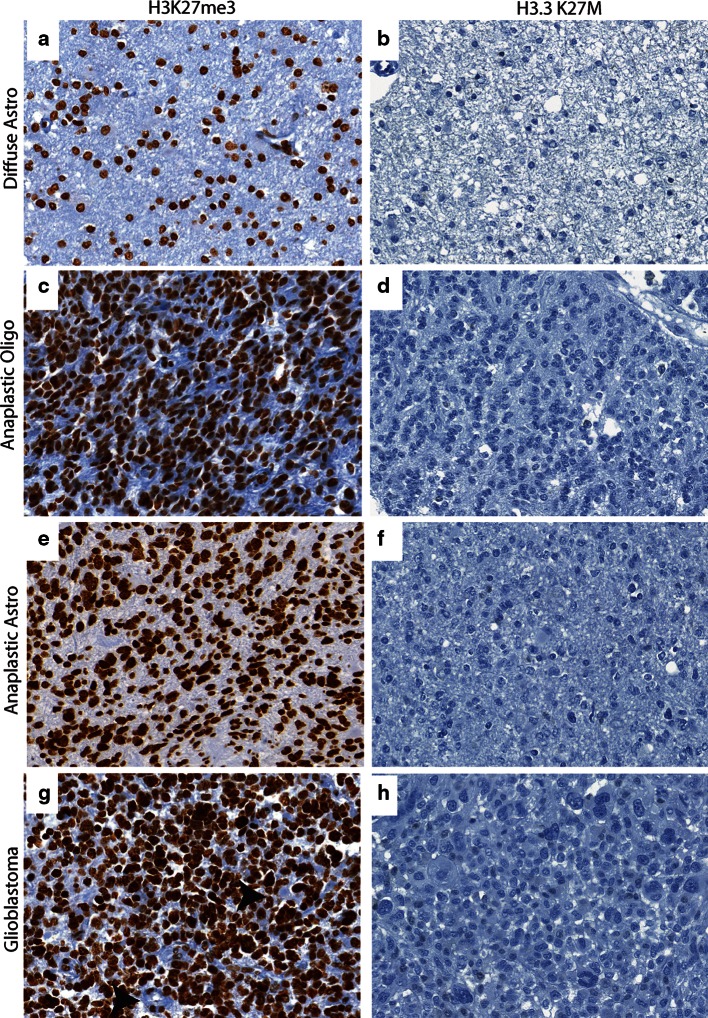
Comparison of H3K27me3 and H3.3 K27M in adult brain tumors. **a**, **b** Representative images of H3K27me3 (40×, **a**) and H3.3 K27M (40×, **b**) in diffuse astrocytoma. **c**, **d** Representative images of H3K27me3 (40×, **c**) and H3.3 K27M (40×, **d**) in anaplastic oligodendroglioma **e and f.** Representative images of H3K27me3 (40×, **e**) and H3.3 K27M (40×, **f**) in anaplastic astrocytoma. **g**, **h** Representative images of H3K27me3 (40×, **g**) and H3.3 K27M (40×, **h**) in adult GBM

**Fig. 6:**
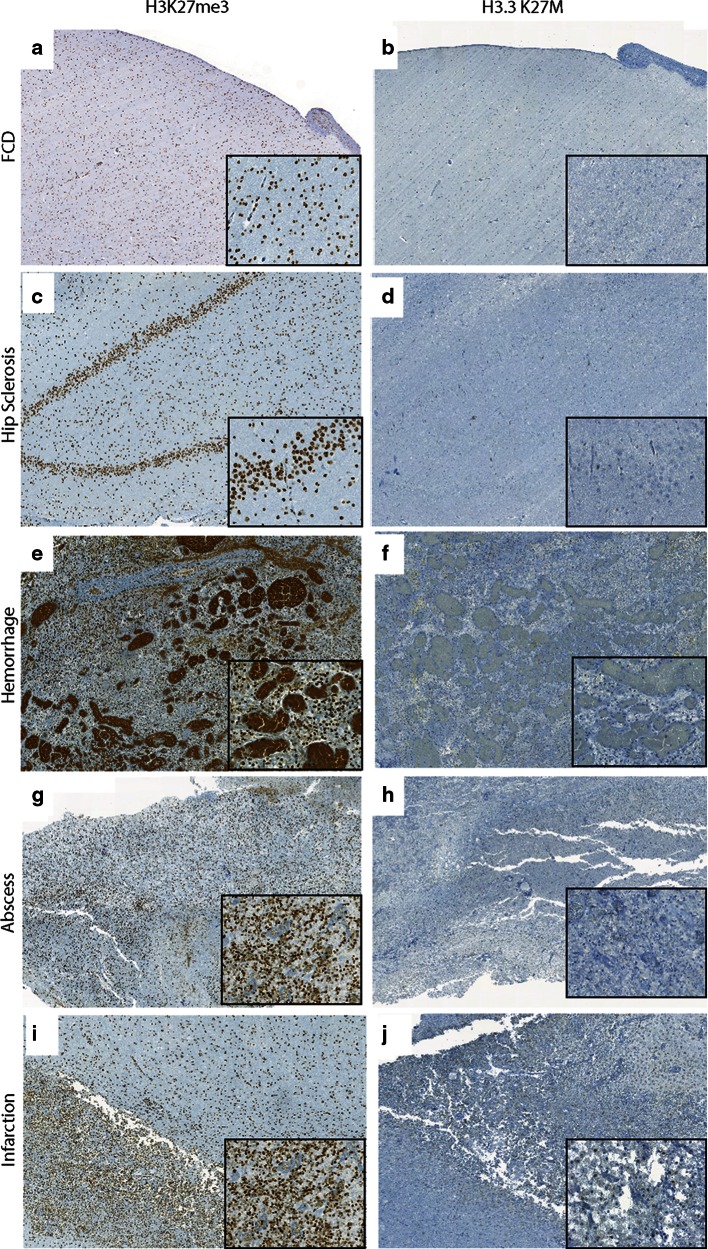
Comparison of H3K27me3 and H3.3 K27M in non-neoplastic brain tissues. **a**, **b** Representative images of H3K27me3 (5×, **a**, *inset* 40×) and H3.3 K27M (5×, **b**, *inset* 40×) in focal cortical dysplasia (FCD). **c**, **d** Representative images of H3K27me3 (10×, **c**, *inset* 40×) and H3.3 K27M (10×, **d**, *inset* 40×) in hippocampal (Hip) sclerosis. **e**, **f** Representative images of H3K27me3 (5×, **e**, *inset* 40×) and H3.3 K27M (5×, **f**, *inset* 40×) in hemorrhage. **g**, **h** Representative images of H3K27me3 (5×, **g**, *inset* 40×) and H3.3 K27M (5×, **h**, *inset* 40×) in abscess, *inset* shows abscess wall. **i**, **j** Representative images of H3K27me3 (5×, **i**, *inset* 40×) and H3.3 K27M (5×, **j**, *inset* 40×) in infarction, *inset* shows macrophages

H3K27me3 was assessed in all tumor samples. Global reduction in H3K27me3 was observed in all 12 *H3F3A* K27M mutant tumors while H3K27me3 was not globally reduced in tumors without the mutation including the *H3F3A* G34R mutant tumor (Figs. 1, 2; Supplementary Figure S1 and Tables S1 and S2). H3K27me3 staining was highest in medulloblastomas (Figs. 2, 3). AT/RT showed a variable pattern where five (5/26) cases showed a reduction in H3K27me3 staining (Fig. 3; Supplementary Figure S2). Relatively lower H3K27me3 values in tumors such as DNET, GG and pediatric pilocytic astrocytomas were not due to reduced H3K27me3 in tumor cells but due to relatively lower cellularity of these tumors (Figure S3). The other pediatric tumors as well as adult brain tumors showed strong nuclear H3K27me3 reactivity (Figs. 4, 5). We also evaluated H3K27me3 in the normal brain and non-neoplastic brain tissues including tissues with infarction, hemorrhage, cortical malformations, hippocampal sclerosis, infections, vascular malformations and hippocampal sclerosis. H3K27me3 was ubiquitously present and showed strong nuclear staining in all cases (Fig. 6).

### H3.3 K27M specific antibody shows positivity in *H3F3A* K27M mutant tumors

We next performed immunohistochemistry using an antibody specifically generated against the N-terminus of the histone mutant H3.3 K27M protein [15] in all tissue samples. H3.3 K27M showed strong nuclear staining in more than 80 % of tumor cells in all *H3F3A* K27M mutant tumors, but not in samples without the mutation (Figs. 1, 2). Endothelial cells within the tumor did not show positivity, suggesting that staining was specific to tumor cells (Fig. 1). Moreover, the case with *H3F3A* G34R mutation did not show any immunoreactivity (Supplementary Figure S1). H3.3 K27M did not show nuclear staining and was significantly lower in the rest of the *H3F3A* wild-type high-grade gliomas and all other pediatric and adult and brain tumor samples and non-neoplastic brain tissues (Figs. 2, 3, 4, 5, 6, S3; Supplementary Table S1). Occasional scattered cells in reactive tissues, other pediatric brain tumors and wild-type cases, showed faint, granular, cytoplasmic staining that was interpreted as non-specific staining as this was similar in non-neoplastic and neoplastic tissues.

Since *H3F3A* K27M mutant tumors are thought to have a worse prognosis than wild-type tumors [3, 13, 18], we evaluated the prognostic utility of the H3.3 K27M antibody. Overall survival was significantly lower in H3.3 K27M positive tumors compared to H3.3 K27M negative tumors (Fig. 2c, Log-rank (Mantel–Cox) test, hazards ratio = 4.97, *p* = 0.015). Multivariate analysis using the Cox proportional hazards model showed that H3.3 K27M positivity was a significant prognostic factor (*p* = 0.025), but not age, sex, grade or location (Fig. 2c; Table 2). These data suggest that immunohistochemical assessment of *H3F3A* K27M mutational status is sufficient for prognostication.

### Comparison of sensitivity and specificity of H3K27me3 and H3.3 K27M positivity in diagnosing *H3F3A* K27M mutant tumors

We compared data from both markers to evaluate sensitivity, specificity, positive predictive and negative predictive values in our tissue samples. Immunohistochemistry for H3.3 K27M was highly sensitive and specific (100 %) with 100 % PPV and NPV (Table 2). Global reduction of H3K27me3, while still sensitive (100 %) and specific (98 %), showed an overall lower PPV of 70 % because of lowered H3K27me3 in 5/26 AT/RT cases (Table 1; Supplementary Table 2), suggesting that H3.3 K27M detection may be the preferable diagnostic biomarker.

**Table 1:** Comparison of H3K27me3 and H3.3 K27M in diagnosing *H3F3A* K27M mutant tumors

Factor	H3.3 K27M positive	H3K27me3 low
Sensitivity	100	100
Specificity	100	98
PPV	100	70
NPV	100	100

**Table 2:** Multivariate Cox regression analysis in pediatric glioblastomas

Variable	*p* value	Hazards ratio	95 % CI for hazards ratio	
			Lower	Upper
Age	0.871	1.006	0.938	1.079
Sex	0.408	1.433	0.611	3.361
Grade	0.932	0.927	0.161	5.324
Location	0.878	0.902	0.243	3.353
H3.3 K27M immunopositive	**0.025**	3.514	1.17	10.559

Age, sex, grade, location, and H3.3 K27M immunopositivity were used as variables in Cox proportional hazards model

H3.3 K27M immunopositivity was a significant prognostic factor (*p* = 0.025) but not age, sex, grade or location

Bold font indicates statistical significance (*p* value below 0.05)

## Discussion

Brain tumors constitute the most common solid malignancy of the pediatric age group. Amongst these, glioblastomas and DIPGs have a dismal prognosis. Recent genomic studies of pediatric high-grade gliomas and DIPGs have revealed mutations in histone variant genes encoding histone proteins, Activin A receptor, type I (ACVR1) and protein phosphatase 1D (PPM1D) bringing new insights into the pathogenesis of these malignancies [4, 8, 13, 17, 18, 20, 24–26]. Histone mutations involve *H3F3A* (encoding histone H3.3) at the K27 and G34 positions in pediatric glioblastomas (~30 %) and DIPG (~80 %) and more rarely *HIST1H3* (encoding H3.1) K27 mutations [4, 9, 10, 13, 17, 18, 24]. Interestingly, *H3F3A* K27M mutations are also noted in thalamic gliomas in young adults [1]. *H3F3A* K27M results in global reduction of H3K27me3 [2, 6, 15, 22]. The mutant K27M inhibits the activity of the PRC2 enzymatic complex by interacting with EZH2, the component of the PRC2 complex that methylates H3K27 [2, 15]. Clinically, these tumors are characterized by worse prognosis, predilection for younger age groups and more frequent midline CNS location [7, 17]. Further, newer experimental therapies targeting enzymes that mediate DNA and histone methylation are also emerging [11]. These factors underscore the urgent and unmet need to identify *H3F3A* K27M mutant tumors in day-to-day surgical neuropathologic practice in a rapid, accurate and cost-effective manner.

To address this, we evaluated two potential biomarkers to recognize *H3F3A* K27M mutant tumors using immunohistochemistry, a tool that is readily accessible to most pathology practices. Recent sequencing analyses of 338 pediatric brain tumors (including many of the varieties of pediatric brain tumors included in our cohort) have shown that histone *H3F3A* K27M mutations occur exclusively in pediatric astrocytic tumors [10]. Thus, we used a comprehensive cohort of pediatric and adult tumor samples and non-neoplastic brain tissues negative controls to assess the specificity and sensitivity of the two tested biomarkers.

First, we assessed the utility of global reduction in H3K27me3 as a means to identify these tumors. Consistent with what we and others have previously reported, we identified a global reduction in H3K27me3 in tumors bearing *H3F3A* K27M mutations compared to tumors without the mutation (Figs. 1, 2) [2, 6, 15, 22]. These data suggest that lowered H3K27me3 may be a potential biomarker. However, we found that 5/26 AT/RT cases also showed global reduction in H3K27me3 resulting in a net positive predictive value of only 70 % (Table 1). Since AT/RT are characterized by *SMARCB1* mutations, but are otherwise genomically silent [14], we compared the expression of EZH2 in cases with or without reduction in H3K27me3. We found that EZH2 expression was lower in AT/RTs with reduced H3K27me3, possibly explaining reduced H3K27me3 in the subset of these tumors observed in this study (Supplementary Figure S2).

To find a better marker, we used a commercially available antibody that specifically targets the H3.3 K27M mutation [15]. This antibody showed nuclear staining specifically in tumor cells (Fig. 1). Endothelial cells within the tumor stained negative for this antibody, serving as an optimal internal control (Fig. 1). We then assessed H3.3 K27M staining in a total of 290 tissue samples including a variety of pediatric and adult brain tumors and non-neoplastic brain tissues. We found that H3.3 K27M staining was restricted to *H3F3A* K27M mutant tumors resulting in 100 % sensitivity, specificity and positive and negative predictive values (Fig. 2; Table 1). Further, immunohistochemistry with H3.3 K27M was sufficient to identify cases with a poor prognosis (Fig. 2; Table 2), suggesting that prognosis determined by immunostaining is very similar to that determined by sequencing studies [13, 18]. Moreover, 66 % (8/12) of tumors positive for H3.3 K27M and bearing *H3F3A* K27M mutations were midline in location compared to 23 % (6/26) of tumors negative for H3.3 K27M and without the mutation (Supplementary Table S1). Difference in age at presentation did not differ significantly between H3.3 K27M positive tumors with *H3F3A* K27M compared to tumors without these mutations.

We present data from a small cohort of pediatric high-grade GBM and a comprehensive set of other tumors and non-neoplastic tissues showing that immunohistochemical staining for H3.3 K27M is a sensitive and specific diagnostic and prognostic biomarker to identify *H3F3A* K27M mutant tumors. While a larger cohort of pediatric high-grade astrocytomas needs to confirm these results, our data demonstrate that immunohistochemistry for H3.3 K27M can be an effective and reliable tool to diagnose these tumors in clinical neuropathology practice.

## Electronic supplementary material

Below is the link to the electronic supplementary material.

Supplementary material 1 (PDF 5577 kb)
