# Supplementary material for: A sensitive and specific histopathologic prognostic marker for H3F3A K27M mutant pediatric glioblastomas
Source: Acta Neuropathol. 2014 Sep 9;128(5):743–53. doi: 10.1007/s00401-014-1338-3 (PMC4201755; doi:10.1007/s00401-014-1338-3)
Supplement: Supplementary file 1 — Supplementary material 1 (PDF 5577 kb) [file 401_2014_1338_MOESM1_ESM.pdf]

Supplementary Information

A sensitive and specific histopathologic prognostic marker for *H3F3A* K27M mutant pediatric glioblastomas.

Sriram Venneti[1*], Mariarita Santi[2], Michelle Madden Felicella[3], Dmitry Yarilin[4], Joanna J. Phillips[4] Lisa M Sullivan[2], Daniel Martinez[2], Arie Perry[5], Peter W. Lewis[6], Craig B. Thompson[1], Alexander R. Judkins[7*]

Figure S1

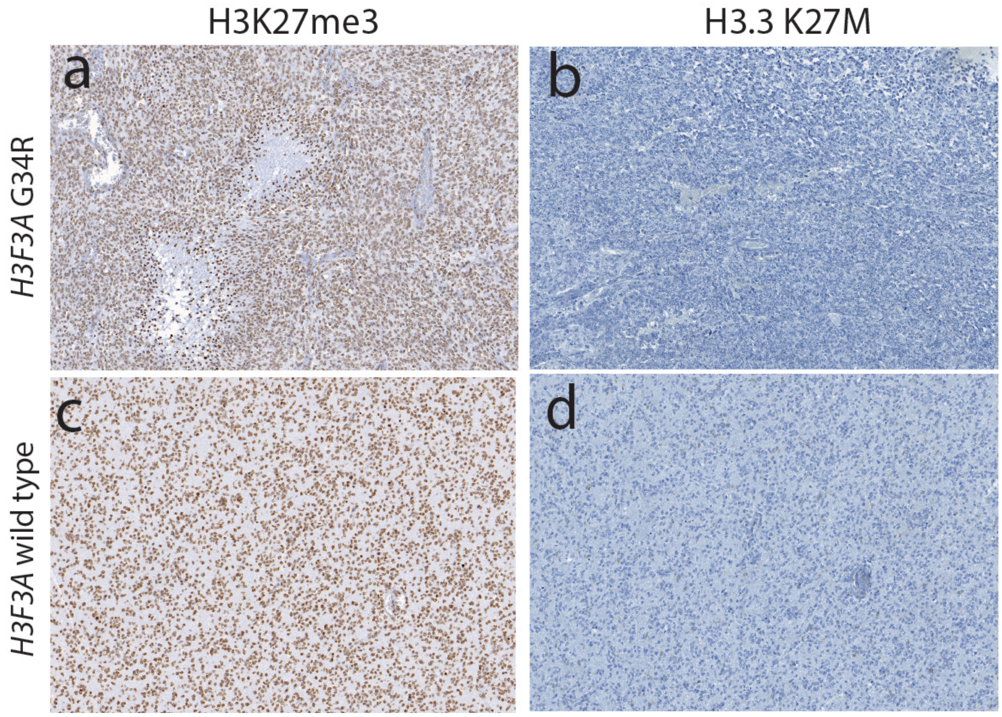

Figure S1.

Figure 1. Comparison of H3K27me3 and H3.3 K27M in *H3F3A* G34R mutant and wild type pediatric GBM.

a and b. Representative images from a *H3F3A* G34R mutant tumor, H3K27me3 (20X, a) and H3.3 K27M (20X, b).

c and d. Representative images from a *H3F3A* K27M wild type tumor, H3K27me3 (20X, c) and H3.3 K27M (20X, d).

Figure S2

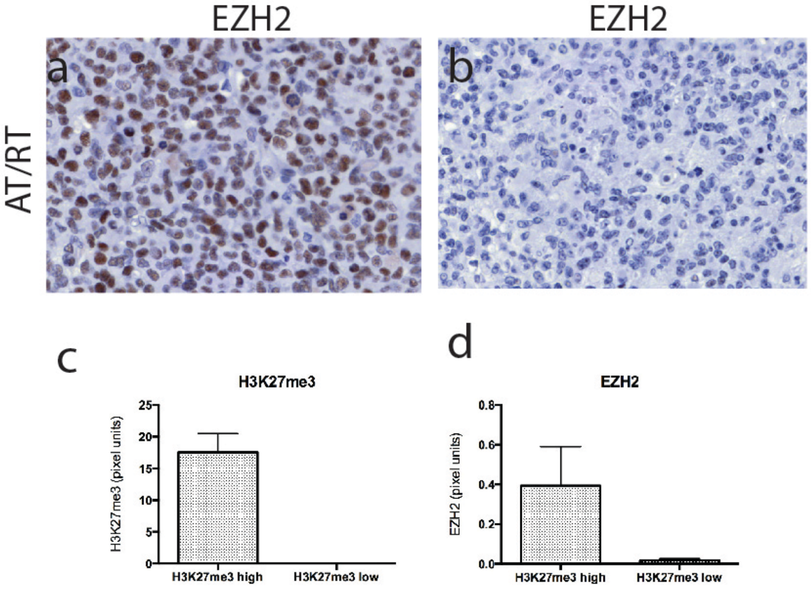

Figure S2. Differences in expression of EZH2 relate with differences in H3K27me3 in AT/RT.

- **a.** Representative image (40X) from an AT/RT case that showed high H3K27me3 and high EZH2 expression.
- **b.** Representative image (40X) from an AT/RT case with low H3K27me3 and low EZH2 expression
- **c.** Quantification of H3K27me3 in cases with high (n=21) and low (n=5) H3K27me3.
- **d.** Quantification of EZH2 in same cases with high (n=21) and low (n=5) H3K27me3.

Figure S3

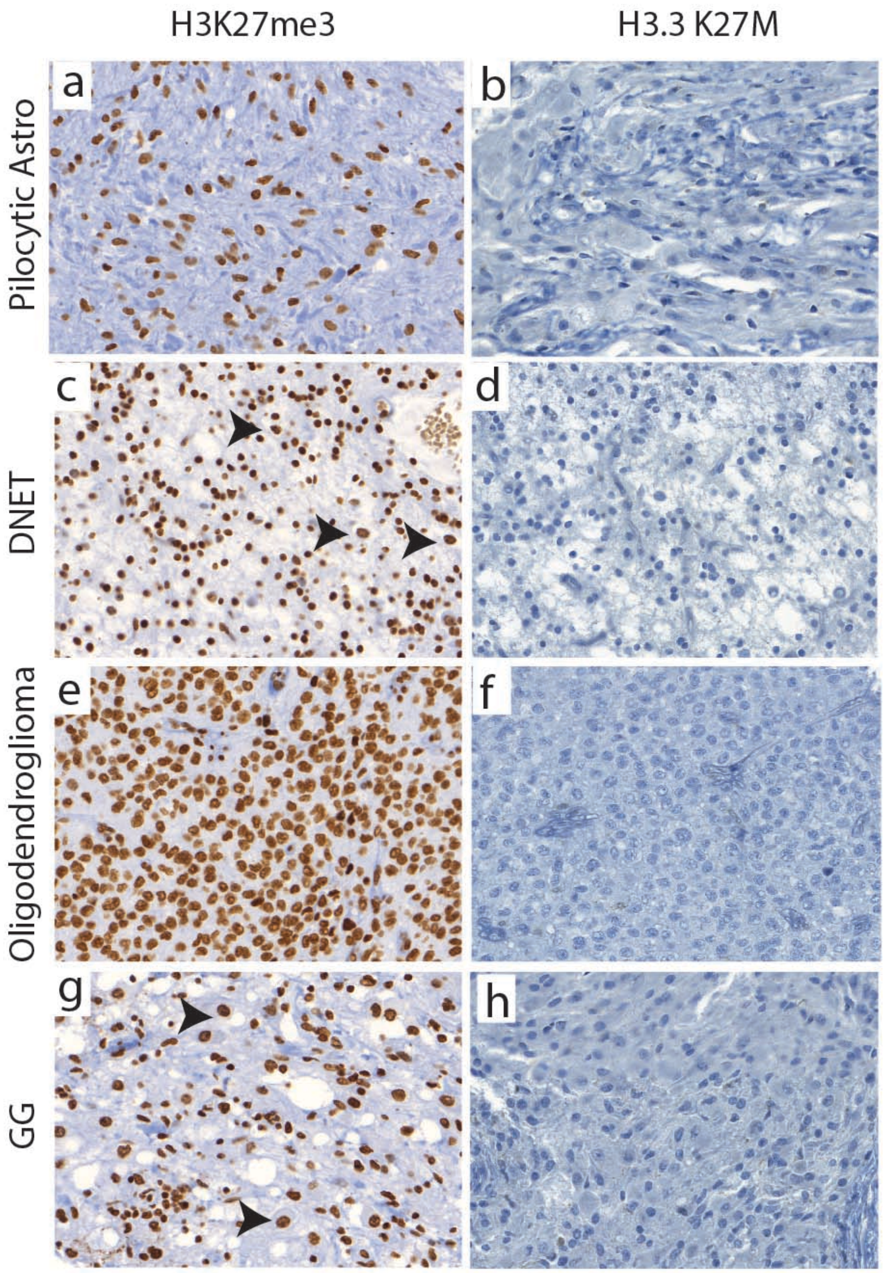

Figure S3. Comparison of H3K27me3 and H3.3 K27M in low-grade pediatric gliomas and glioneuronal tumors.

a and b. Representative images of H3K27me3 (40X, a) and H3.3 K27M (40X, b) in pilocytic astrocytoma

c and d. Representative images of H3K27me3 (40X, c) and H3.3 K27M (40X, d) in DNET (arrowheads indicate floating neurons).

e and f. Representative images of H3K27me3 (40X, e) and H3.3 K27M (40X, f)

in oligodendroglioma

g and h. Representative images of H3K27me3 (40X, g) and H3.3 K27M (40X, h) in ganglioglioma (GG, arrowheads indicate ganglion cells).

Table S1

					Sanger sequencing		Immunohistochemistry	
**Case**	**Diagnosis**	**Sex**	**Age (years)**	**Location**	***H3F3A* K27M**	***H3F3A* G34**	**H3.3 K27M +ve**	**H3K27me3 low**
1	GBM	M	8	Cerebrum	K27M	no	Pos	Yes
2	GBM	M	12	Spinal cord, NOS	K27M	no	Pos	Yes
3	GBM	M	4	Frontal lobe	K27M	no	Pos	Yes
4	GBM	F	7	Thalamic	K27M	no	Pos	Yes
5	GBM	F	15	Bithalamic	K27M	no	Pos	Yes
6	GBM	M	12	Right Thalamus	K27M	no	Pos	Yes
7	GBM	F	21	Thalamus	K27M	no	Pos	Yes
8	AA	M	11	Left thalamus	K27M	no	Pos	Yes
9	GBM	M	9	Right cerebellum	K27M	no	Pos	Yes
10	GBM	F	10	Left occipital lobe	K27M	no	Pos	Yes
11	GBM	F	14	Spinal Cord	K27M	no	Pos	Yes
12	GBM	M	9	Cerebrum, NOS	K27M	no	Pos	Yes
13	GBM	F	0	Cerebrum, NOS	no	G34R	Neg	No
14	GBM	M	32	Spinal cord, NOS	no	no	Neg	No
15	GBM	M	1	Lateral ventricle	no	no	Neg	No
16	GBM	F	18	Frontal	no	no	Neg	No
17	GBM	F	18	Supratentorial, NOS	no	no	Neg	No
18	GBM	M	15	Frontal lobe	no	no	Neg	No
19	GBM	M	16	Brainstem, cbl peduncle	no	no	Neg	No
20	GBM	M	4	Parietal lobe	no	no	Neg	No
21	GBM	F	5	Frontal lobe	no	no	Neg	No
22	GBM	F	8	Temporal lobe	no	no	Neg	No
23	GBM	M	7	Right Temporal	no	no	Neg	No
24	GBM	M	17	Right Temporal	no	no	Neg	No
25	GBM	M	8	Left temporal	no	no	Neg	No
26	GBM	F	17	Left Post Temporal	no	no	Neg	No
27	GBM	F	26	Temporal lobe	no	no	Neg	No
28	AA	M	7	Right temporal lobe	no	no	Neg	No
29	AA	F	14	Right thalamus	no	no	Neg	No
30	AA	M	13	Right temporal	no	no	Neg	No
32	GBM	F	7	Left thalamus	no	no	Neg	No
33	GBM	M	23	Pineal/third ventricle	no	no	Neg	No
34	GBM	M	16	Right temporal lobe	no	no	Neg	No
35	GBM	F	31	Cerebrum , NOS	no	no	Neg	No
36	GBM	M	3	Right temporal lobe	no	no	Neg	No
37	GBM	F	16	Cerebellum	no	no	Neg	No
38	GBM	F	9	Right parieto-occipital	no	no	Neg	No

Table S1. Summary of demographics, sequencing, H3K27me3 and H3.3 K27M staining in all pediatric high-grade astrocytomas.

Table S2

Peditaric brain tumors	n	H3K27me3 low	H3.3 K27M +ve
PA	36	0	0
SEGA	5	0	0
Astrocytoma	16	0	0
OD	5	0	0
DNET	9	0	0
GG	17	0	0
MB	24	0	0
CPP	11	0	0
CP	3	0	0
NC	3	0	0
Men	10	0	0
AA *H3F3A* WT	2	0	0
AA H3F3A K27M	1	1	1
GBM *H3F3A* WT	24	0	0
GBM H3F3A K27M	11	11	11
AT/RT	26	5	0
**Total**	**203**	**17**	**12**
Adult brain tumors	n	H3K27me3 low	H3.3 K27M +ve
PA	4	0	0
GG	2	0	0
DA	3	0	0
AA	8	0	0
OD	3	0	0
AO	5	0	0
GBM	13	0	0
**Total**	**38**	**0**	**0**
Non-neoplastic tissues	n	H3K27me3 low	H3.3 K27M +ve
Normal brain	3	0	0
Vascular malformations	4	0	0
Ischemia	4	0	0
Hemorrhage	5	0	0
CNS malformations	5	0	0
Hippocampal sclerosis	5	0	0
Vasculitis	5	0	0
Demyelinating	5	0	0
Infectious	8	0	0
Metastatic	5	0	0
**Total**	**49**	**0**	**0**

Table S2. Summary of H3K27me3 and H3.3 K27M staining in all tissue samples.
